# Reduced subcutaneous adipogenesis in human hypertrophic obesity is linked to senescent precursor cells

**DOI:** 10.1038/s41467-019-10688-x

**Published:** 2019-06-21

**Authors:** Birgit Gustafson, Annika Nerstedt, Ulf Smith

**Affiliations:** The Lundberg Laboratory for Diabetes Research, Department of Molecular and Clinical Medicine, the Sahlgrenska Academy at the University of Gothenburg, Gothenburg, SE41345 Sweden

**Keywords:** Medical research, Molecular medicine

## Abstract

Inappropriate expansion of the adipose cells in the subcutaneous adipose tissue (SAT) is a characteristic of hypertrophic obesity and of individuals with genetic predisposition for T2D (first-degree relatives; FDR). It is associated with insulin resistance, a dysfunctional, adipose tissue and reduced adipogenesis. We examined the regulation of adipogenesis in human SAT precursor cells and found ZNF521 to be a critical regulator of early adipogenic commitment and precursor cells leaving the cell cycle. However, neither altered upstream signalling nor lack of SAT progenitor cells could explain the reduced adipogenesis in hypertrophic obesity. Instead, we show that progenitor cells undergoing poor differentiation are characterized by senescence, inability to suppress p53/P16^INK4^ and secretion of factors reducing adipogenesis in non-senescent cells. We found aging, FDR and established T2D to be associated with increased progenitor cell senescence, reduced adipogenesis and hypertrophic expansion of the SAT adipose cells.

## Abstract (web summary)

Adipose tissue hypertrophy in obesity is associated with insulin resistance and other metabolic complications. Here, the authors analyze subcutaneous adipose tissue from patients with hypertrophic obesity and insulin resistance and find that adipocyte progenitor cells show features of senescence and have poor differentiation capacity.

## Introduction

Subcutaneous adipose tissue (SAT) is the largest and best storage site of excess fat in the body provided that new cells can be recruited as needed (hyperplastic obesity). Inappropriate expansion of the adipose cells (hypertrophic obesity) promotes insulin resistance and other obesity-associated metabolic complications and is a consequence of inability to recruit new adipose cells. This has been shown both in vitro and in direct studies of human adipose cell turnover in vivo^1–3^.

Our previous extensive studies^2–4^ have shown large inter-individual differences in ability of human SAT stromal vascular fraction (SVF) cells to undergo adipogenesis. Furthermore, markers of reduced SAT adipogenesis are associated with genetic predisposition for type 2 diabetes (T2D) and first-degree relatives (FDR), like individuals with manifest T2D, are characterized by hypertrophic obesity^4–6^. FDR exhibit an obese metabolic profile with insulin resistance and dyslipidemia even when non-obese^5,7^. Recent large genetic studies have also shown that individuals carrying risk genes for T2D and insulin resistance are characterized by reduced SAT^8^.

Human SAT contains a pool of adipose progenitor cells but the detailed signals for recruiting new adipose cells and the reasons for the large individual differences are unclear^1,9^. Bone morphogenetic protein 4 (BMP4) is important for the commitment of mesenchymal progenitor cells into the adipogenic lineage^2,10^ and BMP-signalling is regulated by different secreted inhibitors. We found Gremlin-1 (GREM1) to be an important BMP4 antagonist and increased in hypertrophic obesity^2^. Thus, impaired commitment of progenitor cells could be one reason for the reduced adipogenesis.

Other important regulators of early adipogenesis include the zinc finger proteins (ZNF) 423 and 521. ZNF521 has been suggested to act as a switch for progenitor cell commitment to either the adipogenic or osteogenic lineage^11^ while ZNF423 is a BMP4-regulated activator of peroxisome proliferator-activated receptor γ (PPARG) transcription^12,13^ and important for maintaining the white adipose cell phenotype.

Cell senescence is, in part, a consequence of repeated progenitor cell mitogenic expansion and there is a 10% annual cell turnover in human SAT^9^. Cell senescence leads to permanent cell cycle arrest, secretion of different senescence-associated proteins and inhibited cell differentiation. It is induced by telomere-dependent or independent DNA damage but also by cellular stress including inflammation and oxidative stress^14,15^.

We here characterize mechanisms for the impaired SAT adipogenesis in adult human subjects with hypertrophic obesity including FDR/T2D. We conclude that number of adipogenic progenitor cells is not reduced in hypertrophic obesity. Examination of early adipogenic signals identifies ZNF521 as a key regulator of maintaining human adipose SVF cells proliferative and uncommitted. Silencing ZNF521 increases BMP4 and nuclear import of the adipogenic marker and PPARγ transcriptional activator ZNF423. However, a key mechanism for the impaired adipogenesis in hypertrophic obesity/T2D is increased progenitor cell senescence, dysregulated p53 and P16^ink4^ and secretion of senescence-associated secretory phenotype (SASP) factors antagonizing normal cell adipogenic differentiation.

## Results

### Progenitor cells are not reduced in hypertrophic obesity

**Fig. 1:**
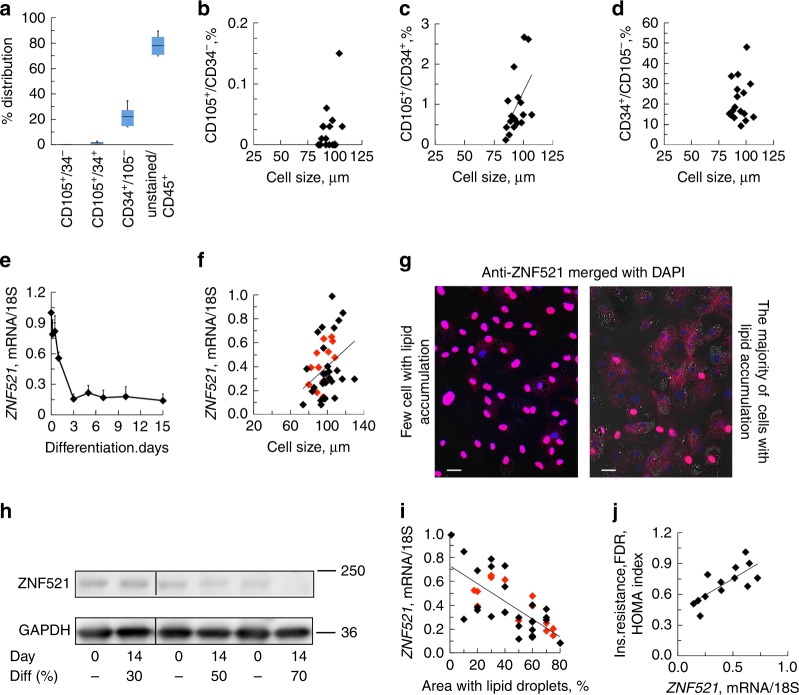
Elevated expression of *ZNF521* in SVF cells is associated with impaired adipogenic differentiation. **a** FACS analysis of the distribution of SVF cells isolated from human subcutaneous adipose tissue. Range CD105^+^/CD34^−^ 0–0.15%, range CD34^+^/CD105^+^, 0.12–2.7%, range CD34^+^/CD105^−^ 9.2–34.7%, and range unstained/CD45^+^ 49.3–89.6%. Data represent ±SEM, *n* = 17 biologically independent samples. **b**–**d** Inter-relationship cell size vs. progenitor cells. **c** CD105^+^/CD34^+^ cells vs. adipose cell size, *P* *=* 0.012. Spearman´s correlation coefficient was used due to not normal distribution. **e** *ZNF521* decreased during differentiation of SVF cells and reached a low plateau at day 3 *n* = 3 independent experiments. **f** Ratio of *ZNF521* expression at differentiation day 14 and day 0 vs. adipose cell size of donor. Association were determined using Pearson correlation analysis *P* *<* 0.01, *n* = 43 biological independent samples. Controls black diamonds and FDR red diamonds. **g** Immunofluorescence of ZNF521 shows that SVF cells that acquire few lipids during differentiation retain ZNF521 in the nucleus. Differentiation day 6, double staining with DAPI. Scale bar 50 µm. **h** Immunoblot of ZNF521 in SVF cells with different degree of differentiation. Immunoblots from the same membrane. Variations at day 0 can be due to committed cells. **i** Correlation between lipid accumulation, and *ZNF521* at day 14. Spearman′s correlation coefficient was used due to not normal distribution, *P* *<* 0.001, *n* = 43 biological independent samples. Individuals from **f**. **j** Association with *ZNF521* and HOMA-IR in FDR. Association were determined using Pearson correlation analysis. *P* *<* 0.01, *n* = 13 biologically independent samples. **f**, **i**, **j** mRNA results were first normalized to 18S and then normalized to expression levels in the undifferentiated sample (=1)

We first examined whether the impaired SAT adipogenesis in hypertrophic obesity is due to reduced number of mesenchymal progenitor/precursor cells. Most cells isolated from fresh SAT stromal vascular fraction (SVF) were CD45^+^ hematopoietic cells (Fig. 1a), followed by CD34^+^/CD105^−^ cells and only few CD105^+^/CD34^−^ mesenchymal stem cells (MSC)^16,17^ (Fig. 1a, and Supplementary Fig. 1a–f and Supplementary Table 1). There was no correlation between adipose cell size or BMI and number of CD105^+^/CD34^−^cells (Fig. 1b) while CD105^+^/CD34^+^ cells were positively correlated with cell size (*P* = 0.012, *n* = 17, Spearman′s correlation coefficient), (Fig. 1c). In agreement with Acosta et al^6^, the frequency of adipose CD34^+^/CD105^−^ progenitor cells were ≈22% and without any correlation with cell size (Fig. 1d). Thus, impaired SAT adipogenesis in hypertrophic obesity is not due to reduced numbers of progenitor cells. We then focused on the role of upstream adipogenic signals/response.

### Repression of ZNF521 is crucial for adipogenesis

**Fig. 2:**
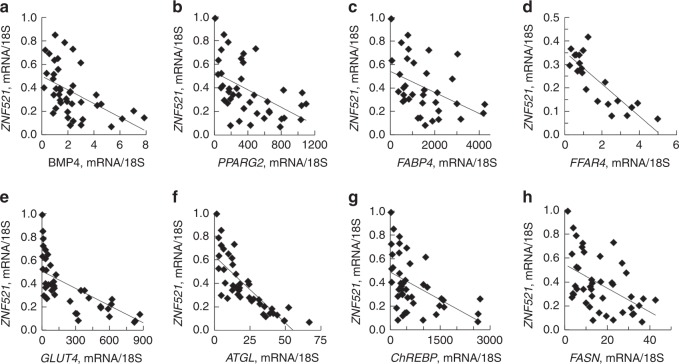
*ZNF521* is a key marker of adipogenic differentiation. **a**–**e** Inter-relationship between expression of the adipogenic markers and *ZNF521*. *FABP4*; *fatty acid binding protein 4* and *GLUT4*; *solute carrier family 2 member 4* (*SLC2A4*). **f**–**h** Inter-relationship between expression of the lipid markers *palatin like phospholipase domain containing 2* (*PNPLA2* also known as *ATGL*), *MLX interacting protein like* (*MLXIPL* also known as *ChREBP*) and *FASN*. **a**–**h** mRNA results were first normalized to 18S and then normalized to expression levels in the undifferentiated sample (=1). Spearman′s correlation coefficient was used since *ZNF521* was not normally distributed. *FABP4 P* *<* 0.01 *n* = 37 biological independent samples, remaining tested genes *P* *<* 0.001, *n* = 42 biological independent samples

ZNF521 is an early switch in murine progenitor cells for commitment into adipogenesis or osteogenesis^11,18^. *ZNF521* mRNA was highly expressed in undifferentiated SVF cells but there were very large inter-individual differences in the ability to repress *ZNF521* following induction of adipogenesis (range at day 15; 0.07–0.99, *n* = 43, Fig. 1e) consistent with the large inter-individual differences in adipogenesis^2–4^. *ZNF521* mRNA in differentiated SVF cells correlated positively with adipose cell size of the donors further supporting that adipogenesis is reduced in hypertrophic obesity (Fig. 1f). Additionally, SVF cells with poor differentiation had high remaining nuclear localization of ZNF521 (Fig. 1g) and ZNF521 protein decreased in parallel with the ability of the cells to undergo adipogenic differentiation (Fig. 1h) and accumulate lipids (Fig. 1i). This was also examined in cells from FDR with similar results documenting their reduced adipogenesis (Fig. 1i). *ZNF521* mRNA also correlated positively with degree of insulin resistance in FDR as a marker of their reduced SAT adipogenesis and expanded adipose cells (Fig. 1j). Suppression of ZNF521 following SVF adipogenic differentiation was inversely related with *PPARG2* together with markers of de novo lipogenesis, lipolysis, glucose, and lipid uptake (Fig. 2a–h) supporting its validity as a marker of cells undergoing adipogenic differentiation.

### Inhibiting ZNF521 triggers adipogenic commitment

**Fig. 3:**
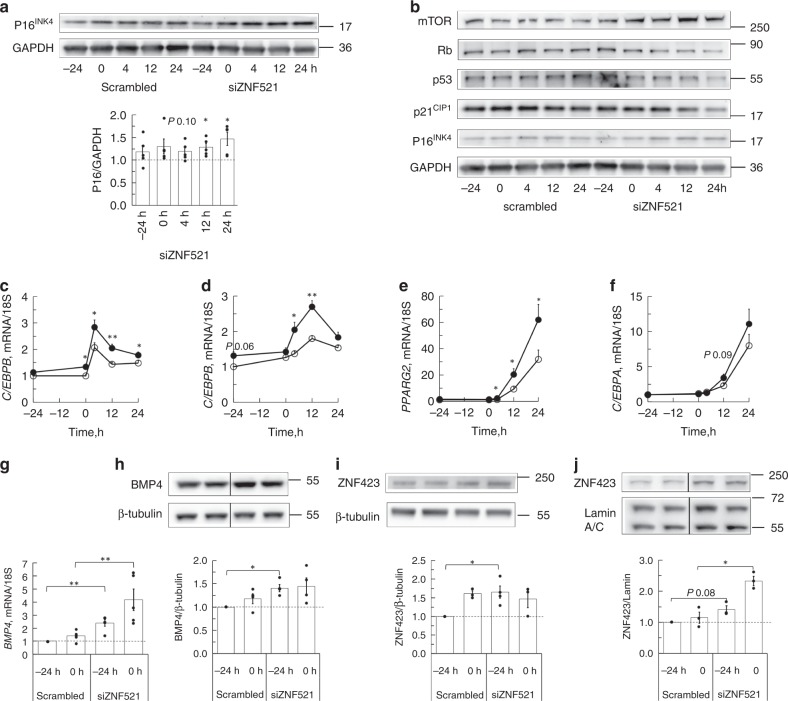
Silencing ZNF521 activates progenitor cell commitment and induces expression of early adipogenic factors. Silencing of ZNF521 was performed 72 h before initiation of differentiation (0 h). **a** Immunoblot of P16^INK4^ after silencing ZNF521. Immunoblots from one individual. Graph shows normalization to scrambled cells at the same time point, *n* = 4 independent experiments, mean ± SEM. Paired two-tailed Student′s *t*-test was used for comparison with scrambled cells. **b** Immunoblots showing cell cycle proteins. Representative immunoblots from one individual of four. **c**–**f** Expression of the early adipogenic genes. Paired two-tailed Student′s *t*-test was used for comparison of silenced with scrambled cells. Data represent means ± SEM *P *<* 0.05 and ** *P* *<* 0.01 *n* = 5 independent experiments. **g** Induction of *BMP4* when ZNF521 is silenced. Data represent means ± SEM, *n* = 5. **g**–**j** mRNA results were first normalized to 18 S and then normalized to expression levels in the undifferentiated scrambled cells at -24 h before initiation of differentiation. **h** Immunoblots of BMP4, *n* = 4 independent experiments and **i** ZNF423 in whole cell lysates, *n* = 4 independent experiments and **j** in nuclear extracts, *n* = 3 independent experiments. **g**–**j** Paired two-tailed Student′s *t*-test was used for comparison of silenced with scrambled cells. *P *<* 0.05 and ** *P* *<* 0.01

ZNF521 was efficiently silenced for 72 h before inducing adipogenic differentiation (Supplementary Fig. 2a). Silencing ZNF521 reduced proliferation of the SVF cells with 30% (±4.5%, *n* = 3), increased their cell size, verified with incorporation of 7-amino-actinomycon D (7-AAD) and FACS analysis (Supplementary Fig. 2b-c) and increased P16^INK4^ (encoded by *CDKN2A*) (Fig. 3a). Additionally, silencing ZNF521 reduced retinoblastoma (Rb), p53 and p21^CIP1^ showing that the cells left the cell cycle (Fig. 3b), and increased the early commitment factors *C/EBPB* and *D* (Fig. 3c, d), which trigger subsequent increase in *PPARG2* and *C/EBPA* (Fig. 3e, f). This can be explained by the increase in BMP4 gene and protein prior to induction of differentiation (Fig. 3g, h) and also associated with increased nuclear import of the PPARG co-activator ZNF423 (Fig. 3i, j). However, silencing ZNF521 was not sufficient to induce general commitment and adipogenesis showing the importance of other overarching inhibitory signals (Supplementary Fig. 2d).

Together, these data show that ZNF521 maintains mesenchymal progenitor cells in an uncommitted and proliferative state. The lack of direct activation of either *C/EBPA* or *PPARG* is consistent with a priming effect of the progenitor cells. We also conclude that the ability to repress *ZNF521* in human progenitor cells is vital for the cells to undergo commitment and subsequent differentiation.

Our findings shed new light on the regulation of human SAT adipogenesis but they do not clearly identify the mechanisms for the impaired adipogenesis and hypertrophic obesity in FDR/T2D^3–6^. We, therefore, examined downstream regulation and the possibility that senescence of the mesenchymal progenitor cells, reported to exist in different aging-associated conditions^14,15^, could be a mechanism.

### Increased cell senescence in adipose SAT biopsies

**Fig. 4:**
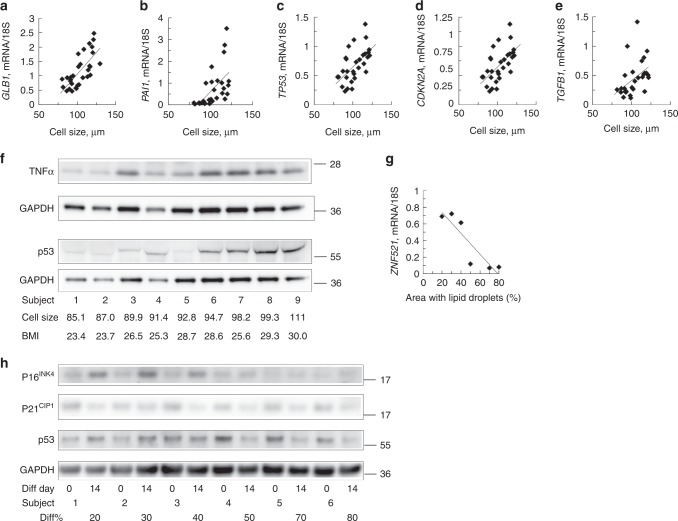
Gene and protein levels of senescence markers are increased in hypertrophic obesity. **a**–**e** Correlation of senescence markers and cell size in fresh SAT biopsies from lean, obese and obese T2D, *GLB1* (*P* *<* 0.001), *PAI1* (*P* *<* 0.001), *TP53* (*P* *<* 0.003), *CDKN2A/P16* ^*INK4*^ (*P* *<* 0.001), and *TGFB1* (*P* < 0.01), *n* = 28 biological independent samples. Comparisons were made with Spearman’s correlation coefficient. **f** Immunoblots of p53 and TNFα in SAT biopsies. **g** *ZNF521* mRNA was reduced in cells with good differentiation measured as area with lipid droplets. Individuals from **h**. **h** Immunoblot from six individuals with different degree of differentiation. SVF cell were differentiated 14 days. **a**–**e** mRNA expression was first normalized to 18 S and then normalized to expression in undifferentiated sample (=1)

To address this, we first examined markers of cell senescence^19,20^ in intact SAT biopsies from 28 individuals with varying BMI and adipose cell size (Supplementary Table 2). As shown in Fig. 4a–e, all senescence markers *β-galactosidase* (*GLB1*), *CDKN2A, plasminogen activator inhibitor-1 (PAI1*, encoded by *SERPINE1)*, *transforming growth factor-β1 (TGFB1)*, and *TP53* (encoding the p53 tumour suppressor) correlated positively with adipose cell size and also with each other (Supplementary Fig. 3a-f). p53, a key regulator of senescence, is increased in fresh SAT tissue biopsies from individuals with hypertrophic obesity (Fig. 4f and Supplementary Fig. 4). These senescence markers in intact SAT tissue were higher in obese vs. lean individuals of similar age (around 38 year in both groups) and further markedly increased in similarly obese T2D. However, the obese T2D individuals were significantly older (61 year vs. 38 year, Supplementary Table 2**)** but increased p53 in the adipose tissue in T2D has also been reported previously^21^. Furthermore, adipose cell size was a much stronger determinant of these markers of senescence than obesity (BMI > 30 kg m^−2^) (Supplementary Table 3).

### Senescent SVF cells do not differentiate to adipose cells

We then examined markers of senescence in SVF cells in relation to their ability to undergo adipogenesis. We selected SVF cells at an early passage from non-obese donors but with different SAT adipose cell size as an in vivo marker of adipogenic response to affluence and characterized them before and after differentiation (BMI range 22.3–29.9 kg m^−2^ and adipose cell size range 73.7–107.4 µm). Individual ability of SVF cells to undergo adipogenesis was characterized as poor (<40%) or good (>50%) with lipid accumulation measured as lipid droplet area. The major differences were seen in P16^INK4^ and its regulator p53; both of which were higher in cells with poor differentiation but showed the expected decrease in cells undergoing good differentiation (Fig. 4h). There was no difference in reduction of p21^CP1^. This dysregulated pattern of increased p53 and P16^INK4^ in poorly differentiating adipose SVF cells was also a characteristic of cells from non-obese FDR (Supplementary Fig. 5) showing that increased progenitor cell senescence is associated with reduced adipogenesis long before T2D develops.

Together, these findings show that SAT adipose precursor cells with poor differentiation are restrained by increased p53 and P16^INK4^ activation^21–23^. This is further validated by high *GLB1* and *ZNF521* as markers of poor differentiation.

This concept was further verified in a larger group of donor cells following adipogenic differentiation showing senescence markers and *TGFB* to be positively correlated with *ZNF521*, and negatively with *PPARG, free fatty acid receptor 4* (*FFAR4*), and lipid droplet accumulation (Supplementary Table 3). *GLB1*, as a highly distinctive senescence marker, showed a strong positive correlation with the senescence markers *PAI1* and *CDKN2A* (Supplementary Table 3).

We also identified *GREM1* to be upregulated in cells with poor differentiation and correlating with markers of senescence (Supplementary Fig. 6). Thus, *GREM1* is an additional likely contributor to the inability of human progenitor cells to undergo commitment and differentiation.

**Fig. 5:**
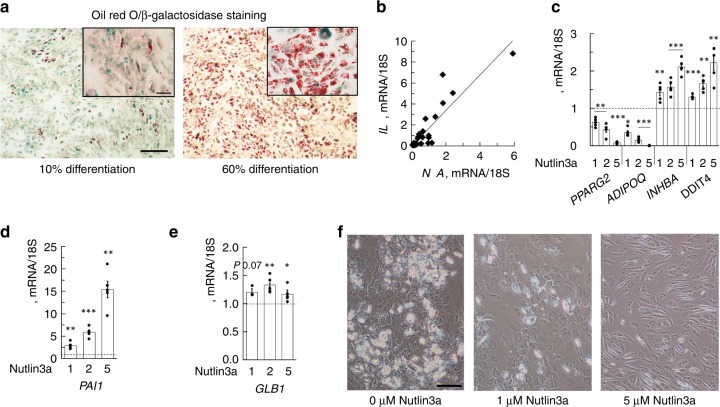
Cell senescence induces proinflammatory signals and reduces lipid accumulation. **a** Double staining of differentiated SVF cells with ORO and β-galactosidase. Result from two individuals with different degree of differentiation. Scale bar 200 µm. **b** *TNFA* vs. *IL6* in whole adipose tissue, *P* *<* 0.001, *n* = 28 biological independent samples. Comparisons were made with Spearman’s correlation coefficient. mRNA expression was first normalized to 18 S and then normalized to expression in undifferentiated sample (=1). **c**–**f** Nutlin3a, inhibitor of p53 degradation, was added to the differentiation cocktail at time 0 h and was present throughout differentiation. Expression of adipogenic and senescence markers. Differentiation for 3 days (**c**–**e**) and 12 days (**f**). Results from five independent experiments, means ± SEM. **f** Images from differentiation day 10 of the cells in figure (**e**). Scale bar 100 µm. **c**–**f** Comparisons were made with Spearman’s correlation coefficient. *P *<* 0.05, **P *<* 0.01, and ***P *<* 0.001

We also examined β-galactosidase^22,23^ in cells with different degrees of differentiation. Cells with good differentiation show no or low β-galactosidase while poor differentiation is associated with many cells exhibiting high intensity. However, also cells with low β-galactosidase can still accumulate some lipids, i.e. undergo partial differentiation (Fig. 5a).

Cell senescence is associated with proinflammatory signals where tumour necrosis factor A (TNFA) is particularly prominent^21,24^. There was also a positive correlation between *TNFA* and *IL6* in SAT (Fig. 5b) and TNFα protein increased with cell size in parallel with p53 protein (Fig. 4f). We also found increased *TNFA* associated with increased DNA damage inducible transcript 4 (*DDIT4*); a marker of regulation of MSC fate and induced by p53 activation (Supplementary Fig. 3g). Thus, inflammation can be a contributor to increased cell senescence in hypertrophic obesity, but our finding of dysregulated P16^INK4^ and p53 in adipose SVF cells from young lean and healthy FDR support other important initiating events involving epi-/genetic regulation restraining adipogenesis. This is consistent with the well-documented associations between T2D risk genes and reduced SAT^8^.

To further verify the importance of p53, SVF cells were differentiated with Nutlin3a, an inhibitor of p53 degradation^25^. Nutlin3a dose-dependently inhibited induction of *PPARG2* and *adiponectin* (*ADIPOQ)*, while markers of senescence including *GLB1* and, most prominently, *PAI1* were increased (Fig. 5c-e). Furthermore, the cells did not accumulate lipids at all (Fig. 5f).

Aging is another important contributor to senescence but we only found *TP53* in the adipose tissue to be positively correlated with age of donors (*P* *<* 0.05, *n* = 31, Spearman′s correlation coefficient) (Supplementary Fig. 7a). This is an interesting finding in itself since p53 in the adipose tissue is an important regulator of whole-body insulin sensitivity^21^ and, thus, may also contribute to the well-established age-associated insulin resistance in man^26^.

Increased progenitor cell senescence with age would also be associated with reduced adipose cell replacement leading to increased cell size with age. We also found adipose cell size to increase significantly with age independent of BMI (*P* *<* 0.023, *n* = 74, Spearman′s correlation coefficient) (Supplementary Fig. 7b). Recent studies have shown age of the donors to be negatively correlated to adipose cell generation^27^ and that amount of visceral fat, a marker of inability to store excess fat in SAT, is positively correlated with age also when compensated for amount of body fat^28^.

Finally, to further identify potential mechanisms whereby senescent cells can regulate adipogenic differentiation, progenitor cells from the same donors were differentiated with conditioned medium (CM) from individuals with markers of cell senescence and defined as undergoing poor (area with lipid droplets < 20%) or good differentiation (>70%). CM from cells with poor differentiation significantly reduced the gene expression of key markers of adipogenic differentiation (*PPARγ2, FABP4, ADIPOQ*, and *GLUT4* encoded by *solute carrier family 2 member 4* (*SLC2A4*)) by a mean of 35% and lipid accumulation was also reduced (Supplementary Fig. 8).

Taken together, our findings show an intricate relation between age, epi-/genetics, and obesity-associated cell environment in regulating adipogenesis and that hypertrophic obesity, a characteristic of FDR/T2D, is associated with increased progenitor cell senescence and reduced adipogenesis. Furthermore, conditioned medium from cells with senescence and poor differentiation reduce adipogenesis of control cells and lead to less lipid accumulation than medium from cells with good differentiation supporting a direct inhibitory role of secreted SASPs on adipogenesis as also previously suggested^29^.

## Discussion

We here focused on the regulation of adipogenesis in human subcutaneous adipose tissue progenitor cells and its association with clinical phenotype. This approach makes translation of cellular data to human phenotype more direct. Furthermore, the importance of senescence as an aging-associated consequence should be particularly important in man considering human longevity for 80–90 years while most murine studies addressing regulation of adipogenesis uses young animals where the situation is quite different from human studies. We reported long ago that the ability of human subcutaneous adipose tissue precursor cells from different individuals to undergo adipogenesis is markedly variable (ranging from 5–10% to over 90% of the cells accumulating lipids) and that inappropriate expansion of the adipose cells, i.e. hypertrophic obesity, is a marker of this^4,30^. Importantly, expanded adipose cells are associated with insulin resistance, enhanced local tissue and systemic inflammation and risk of developing T2D^3^. However, the basic mechanisms for this large variation by adipose precursor cells to undergo adipogenesis have been unclear. The important association between adipogenesis and clinical phenotype was further emphasized by our finding that non-diabetic individuals with a family history of T2D (FDR) are characterized by inappropriate adipose cell hypertrophy^5,31^ while this was not seen in individuals with a family history of obesity^5^. This difference in cell size is particularly seen in non-obese individuals since adipose cells have a similar limited ability to expand in obesity^6^.

In the current study we first asked if the reduced adipogenesis in hypertrophic obesity is a consequence of reduced numbers of stem/progenitor cells but found no such evidence. We have also previously shown that the number of CD133^+^ cells was increased in hypertrophic obesity further supporting a defective ability to differentiate^30^. We initially focused on early upstream signaling and the role of ZNF521, which has been shown to be a switch for the commitment of mesenchymal precursor cells to the osteogenic vs. adipogenic pathway in mice^11^. ZNF521 has also been shown to be an important regulator of the hematopoietic stem cell niche and ability of the cells to proliferate^32^ and has been reported to regulate cell transformation and proliferation of neural stem cells^1^.

We found ZNF521 to indeed be an important regulator of adipose progenitor cells and its silencing reduced cell proliferation and expanded cell size. It is very highly expressed in the precursor cells and cells unable to differentiate retain the high nuclear expression. Thus, ability to reduce ZNF521 during differentiation was strongly positively correlated with number of cells undergoing adipogenic differentiation. We conclude that ZNF521 maintain progenitor cells uncommitted and proliferative and silencing this nuclear factor induces early adipogenic commitment of the cells likely as a consequence of the induction of BMP4, a key regulator of adipogenic commitment, and increased nuclear ZNF423, which is a key regulator of PPARγ transcription and activated by BMP4^1,13^. In separate studies we found that silencing ZNF521 also allowed the cells to undergo excellent osteogenic differentiation (data not shown), which is consistent with an early priming of the progenitor cells rather than promoting a single pathway of differentiation. Indeed, we saw no direct activation of downstream adipogenic differentiation by merely silencing ZNF521.

Although an important regulator of early mesenchymal progenitor cell commitment, dysregulated ZNF521 was not the mechanism for the reduced adipogenesis in hypertrophic obesity. Instead, our data show that progenitor cell senescence plays a key role. Cell senescence has attracted much recent interest as a mechanism for impaired growth and cell replacement in normal aging as well as in several different aging-associated disorders including cardiovascular disease and T2D^33,34^. Very intriguingly, it was recently demonstrated that transplanting senescent cells to mouse models reduced function and life span^35^ probably, at least in part, a consequence of their secretion of SASPs, which inhibit normal cell differentiation and enhance inflammation. In addition, a recent extensive study in obese mouse models showed that reducing senescence either genetically or with senolytic agents also alleviated metabolic and adipose tissue dysfunction and promoted adipogenesis^33^.

We here show that senescence markers are increased in intact adipose tissue from individuals characterized by hypertrophic obesity and that adipose cell size rather than BMI is a key marker of tissue senescence. We also show that progenitor cells from these individuals are characterized by increased senescence and this is associated with an impaired adipogenic potential. Age is one important driver of normal senescence but we also show that a genetic predisposition for T2D, defined as being a high-risk FDR characterized by inappropriate hypertrophic expansion of the mature adipose cells^5,31^, is associated with increased cell senescence. Thus, senescence clearly proceeds hyperglycemia and normal aging since the FDR were fairly young (around 36 year). These data suggest the importance of epi-/genetic factors associated with T2D as promoters of early senescence and reduced adipogenesis. Very little is known about this but *CDKN2A* polymorphisms are associated with both increased cardiovascular disease (CVD) and T2D^36^. However, in our genome-wide association studies (GWAS) data on around 250 individuals we have not seen any association between *CDKN2A* SNPs and adipose cell size (data not shown). The only genetic polymorphism shown to be associated with adipose cell size is *KLF14*^37^. However, this is exclusively seen in females and we did not see any significant correlations with *KLF14* mRNA levels in our subjects (data not shown).

p53 needs to be inhibited to allow normal adipogenesis and we also showed that inhibiting reduction of p53 with the E3 ubiquitin ligase inhibitor nutlin-3 completely inhibited adipogenic differentiation of the progenitor cells. Both p53 and its target P16^INK4^ remained increased and were clearly dysregulated in progenitor cells from individuals with hypertrophic obesity and with FDR. Increased p53 has also previously been shown in adipose tissue from individuals with T2D and overexpression of p53 in the adipose tissue of mouse models promotes insulin resistance and inflammation^21,38^. A key question to address is the mechanisms for the dysregulated p53 and its inability to undergo inhibition. One possibility is that signals to activate mouse 3T3 cell double minute 2 homolog (MDM2) are inappropriate and this is under current investigation. The role of inflammation is also important to clarify and it is likely that it contributes to enhancing senescence in the adipose tissue cells in obesity. However, this would make inflammation a secondary phenomenon and not a primary mechanism for early inhibited adipogenesis as seen in FDR.

Taken together, our findings show that ZNF521 is a key regulator of mesenchymal progenitor cells and maintains them in a proliferative and uncommitted state. However, the impaired adipogenesis associated with hypertrophic obesity and genetics for T2D cannot be accounted for by a dysregulated inhibition of ZNF521 but rather by the presence of senescent progenitor cells. These cells cannot undergo proliferation and differentiation but they also secrete SASPs, which are antagonistic to ambient cells. Identifying factors contributing to the dysregulated p53 in adipose progenitor cells can open up novel ways of targeting insulin resistance and prevent CVD and T2D.

## Methods

### Human subjects

Gene expression and protein levels were studied in isolated stromal cells from human abdominal subcutaneous adipose tissue (needle biopsies) from 44 individuals. The subjects were between 22 and 69 years of age and had a mean body mass index of 27.7 ± 3.3 kg m^−2^ (range 22.3–36.1) and adipose cell size 98.3 ± 11.7 µm (range 73.7–129.6). Twenty-nine were healthy control individuals with no known chronic diseases and fifteen were T2D and first-degree relatives (FDR). For studies in fresh adipose biopsies, 33 individuals were included. The subjects were between 26 and 67 years of age and had a mean body mass index of 29.5 ± 13.1 kg m^−2^ (range 20.9–39.5) and adipose cell size 102.3 ± 13.2 µm (range 78.0–121.8). During analysis four individuals were excluded due to bad RNA quality. HOMA-IR was calculated using the HOMA calculator (Radcliff, Dep. of Medicine, University of Oxford, UK).

### Isolation of stromal cells from adipose tissue biopsies

Abdominal subcutaneous adipose tissue biopsies, estimated weight 300–700 mg, were obtained in local anaesthesia. Whole tissue was used for lysates and RNA, isolated adipose cells for cell size and SVF for cell culture. The biopsies were washed with PBS, cut into small pieces and digested with 0.8 mg/mL collagenase (Collagenase A, Roche Diagnostics) in M199 (Medium 199, with Hepes, and amino acids, without glucose, GIBCO) supplemented with 4% BSA, 4 mM NaHCO_3_ (Merck), 5.6 mM glucose, and 150 nM adenosine (Sigma–Aldrich, pH 7.4) for 50 min at 37 °C in a shaking water bath. The digest was filtered through nylon mesh with a pore size of 250 µm. The cells were washed in DMEM supplemented with 10% (v/v) FBS, 2 mM L-glutamine, and antibiotics (GIBCO). The layer with floating adipocytes was removed and used for adipose cell size measurement while the remaining media, containing the stromal fraction, was centrifuged for 15 min at 1500 × *g* at 20 °C. The stromal cells were seeded in a 175 cm^2^ cell culture flask with DMEM supplemented with 10% FBS, glutamine and antibiotics or used for flow cytometry (FACS) analysis.

### BrdU and 7-AAD staining and FACS analysis

Staining of cells was done with the BrdU Flow Kits (BD 552598) according to the manufacturer’s instructions. The cell-associated BrdU and 7-AAD were then measured by flow cytometry (FACS). Erythrocytes were first removed from the SVF with lysing solution (BD 555899) according to the protocol. For analysis, the cells were stained with the fluorophore-conjugated antibodies: PerCP-5.5 labelled anti-CD105 (1:40, BD 560819), APC labelled anti-CD34 (1:20, BD 560940), and FITC labelled anti-CD45 (1:20, BD 560976). As controls, the following isotype antibodies were used: PerCP-Cy 5.5 (1:40, BD 550795), APC (1:20, BD 555751), and FITC (1:20, BD 555748). After staining, the cells were used for FACS analysis (CytoFLEX, Beckman Coulter). Control gates were set based on staining with matched labelled isotype control IgG antibodies. For each experiment, 10,000 cells were counted.

### Adipogenic differentiation of human adipose stromal cells

The isolated stromal cells were expanded in culture using DMEM cell culture medium with 10% FBS. Cells at passage three to four were induced to differentiate, after 3 days of confluence (=day 0), with a cocktail consisting of 850 nM insulin, 10 μM dexamethasone, 0.5 mM isobutylmethylxanthine (IBMX, Sigma–Aldrich), 10 μM pioglitazone (Cayman Chemical) in DMEM supplemented with 3% FBS (v/v), 2 mM L-glutamine, and antibiotics. After 3 days, the medium was changed to adipocyte medium (DMEM with 10% FBS) containing 850 nM insulin, 1 µM dexamethasone, 1 µM pioglitazone, 10% FBS, glutamine, and antibiotics. The adipocyte medium was changed every third day throughout the differentiation period. To examine lipid accumulation, cells were fixed with 10% formalin for 20 min and stained with Oil Red O (ORO). When indicated 1, 2, and 5 µM of Nutlin3a (Sigma–Aldrich SML0580) was added.

### Conditioned medium (CM)—collection and differentiation

Medium from individuals with cells undergoing poor or good differentiation were collected both before differentiation (day 0) as well as at differentiation days 9 and 12. Twenty-four hours before initiation of differentiation, cells from the same donors were incubated with standard medium mixed with day 0 CM at a 1:1 ratio. Differentiation medium was then mixed with CM at a ratio of 1:1 of the pooled days 9 and 12 CM and the cells were differentiated for 12 days. Medium was changed every third day.

### Whole cell extracts and western blots

Protein lysates were extracted in ice-cold lysis buffer (25 mM Tris-HCl, pH 7.4, 0.5 mM EGTA, 25 mM NaCl, 1% Nonidet P-40, 1 mM Na3VO4, 10 mM NaF, 0.2 mM leupeptin, 1 mM benzamidine, and 0.1 mM 4^−^(2-aminoethyl)-benzenesulfonylfluoride hydrochloride). Protein content was determined using BCA Protein Assay kit (Pierce). Proteins were subjected to SDS-PAGE and transferred to nitrocellulose membranes. The membranes were probed with antibodies, ZNF521 (1:750) Sigma–Aldrich, SAB3500840; GAPDH (1:3000) Santa Cruz sc-47724; P16^INK4^ (1:500) Abcam 201980; mTOR (1:500) Cell Signaling 4517; Rb (1:500) Santa Cruz sc-50; p53 (1:500) Cell Signaling 2524; p21^CIP1^ (1:500) Santa Cruz sc-6246; BMP4 (1:1000) Abcam 39973; β-tubulin (1:2500) Cell Signaling 2128; ZNF423 (1:500) Santa Cruz sc-48785; Lamin A/C (1:500) Santa Cruz sc-6215; TNFα (1:500) Cell Signaling 6945; Horse anti-mouse IgG, (1:2000) Cell signaling 7076; Goat anti-rabbit IgG (1:2000) Cell signaling 7074; Donkey anti-goat IgG (1:1000) Santa Cruz sc-2020. A summary of antibodies used is found in Supplementary Table 4. For development ChemiDoc Imaging System (Bio-Rad) was used. Uncropped and unprocessed scans with ladders are shown in the Source Data file.

### Nuclear extracts and small interfering RNA (siRNA)

Nuclear extracts were performed with NE-PER Nuclear and Cytoplasmic Extraction Reagents (Thermo Scientific #78833) according to the manufacturer´s instructions. Human isolated SVF cells were transfected with ZNF521 siRNA (Hs01_00160534, Sigma–Aldrich) or siRNA universal negative control (Sigma SIC001) using RNAiMAX (Thermo Fisher Scientific) according to the manufacturer’s instructions.

### RNA extraction and Q-PCR

Total RNA was isolated from the cells with EZNA total RNA kit (Omega Bio-tek). RNeasy Lipid Tissue Kit was used for isolation of total tissue RNA (Qiagen). Quantification of RNA was performed with NANODROP 1000 Spectrophotometer (Thermo Fisher Scientific). cDNA synthesis was performed with the High Capacity cDNA kit (Thermo Fisher Scientific) according to the manufacturer’s recommendations. Gene expression was analysed with the Quant Studio6 sequence detection system (Thermo Fisher Scientific). Gene-specific primers and probes were designed using Primer Express software (Thermo Fisher Scientific) or purchased as Assay-on-Demand (Thermo Fisher Scientific). Probe and primer sets are listed in Supplementary Table 5. Relative expression was calculated using the ΔΔCt method with normalization to 18S (endogenous control).

### Immunohistochemistry

Human adipose tissue stromal cells were transfected with either siZNF521 or scrambled RNA and grown on glass slides (ChamberSlide, NUNC). Differentiation was initiated as above. Cells were fixed with 4% formaldehyde for 15 min and permeabilized in 0.1% Triton X-100 for 5 min. Cells were then blocked with 20% FBS for 30 min followed by incubation with anti-ZNF521 antibody (1:50, Sigma–Aldrich, HPA023056) for 3 h. After washing in PBS and incubation with secondary antibody conjugated with Alexa-594 (Molecular Probes) for 1 h, the slide was mounted with VectraShield mounting solution (Vector Laboratories). Images were collected either with Zeiss AxioCam MRM or NIKON Eclipse 100 microscope cameras.

### β-galactosidase staining

Cells were plated in a 12-wells plate and fixed in a mixture of glutaraldehyde and formaldehyde for 15 min and stained over-night with X-Gal solution using a commercial kit from Cell Signaling (9860) according to the manufacturer’s description.

### Statistical analysis

The experimental data are presented as means ± SEM of at least three independent experiments as indicated in the Figure legends. Data analysis was performed with PASWstatistics (SPSS Inc.) for Macintosh. Unless otherwise stated, single comparisons with basal samples were performed using two-tailed Student’s paired *t-*test. All results were tested for normal distribution. Spearman′s rank correlation was used to analyse data that were not normally distributed as indicated in the Figures. Two-tailed and unpaired tests were used for data analysis. Differences were considered statistically significant at *P* *<* 0.05 level.

### Study approval

The Ethical Committee of the University of Gothenburg approved the study design and it was performed in agreement with the Declarations of Helsinki. Written informed consent was received from participants prior to inclusion in the study.

### Reporting summary

Further information on experimental design is available in Reporting Summary linked to this article.

## Supplementary information

Supplementary Information

Reporting Summary

Source Data

## Data Availability

Data presented in this manuscript are included in the paper or its supplementary information as the Source Data file or available from the corresponding author upon reasonable request.
