# Supplementary material for: Reduced subcutaneous adipogenesis in human hypertrophic obesity is linked to senescent precursor cells
Source: Nat Commun. 2019 Jun 21;10:2757. doi: 10.1038/s41467-019-10688-x (PMC6588633; doi:10.1038/s41467-019-10688-x)
Supplement: Supplementary file 1 — Supplementary Information [file 41467_2019_10688_MOESM1_ESM.pdf]

Supplementary information

Reduced subcutaneous adipogenesis in human hypertrophic obesity is linked to senescent precursor cells

Birgit Gustafson, Annika Nerstedt and Ulf Smith

Supplementary Fig. 1. Immunophenotypic analysis of SVF cells isolated from fresh human subcutaneous adipose tissue biopsies.

Supplementary Fig. 2. Immunofluorescence staining of incorporated 7-amino-actinomycin D (7-AAD) and FACS analysis of ZNF521 silenced cells.

Supplementary Fig. 3. Correlation between selected senescence markers in SAT biopsies and differentiated SVF cells.

Supplementary Fig. 4. Correlation of p53 and TNFα with cell size in intact adipose tissue biopsies.

Supplementary Fig. 5. Increased susceptibility to develop cell senescence is linked to retained p53 and $P16^{INK4}$ levels in differentiated adipocytes in FDR.

Supplementary Fig. 6. The BMP4 antagonist *GREM1*, highly expressed in cells with poor adipogenesis, correlated with markers of senescence.

Supplementary Fig. 7. Donor age is correlated both with *TP53* and SAT cell size adjusted for BMI.

Supplementary Fig. 8. Conditioned medium from individuals with poor differentiation lead to reduced adipogenesis compared to conditioned medium from cells with good differentiation.

Supplementary Table 1. Frequencies of progenitor/precursor cells subsets in subcutaneous adipose tissue biopsies.

Supplementary Table 2. Biometric data and expression of genes related to senescence of subjects in Figure 4.

Supplementary Table 3. Expression of senescence markers in differentiated adipocytes and their correlations with BMI and markers of adipogenesis.

Supplementary Table 4. Antibodies used for Western blot, immunofluorescence and FACS analysis.

Supplementary Table 5. Probes and primers used for qPCR.

Supplementary Fig. 1, Related to Fig. 1

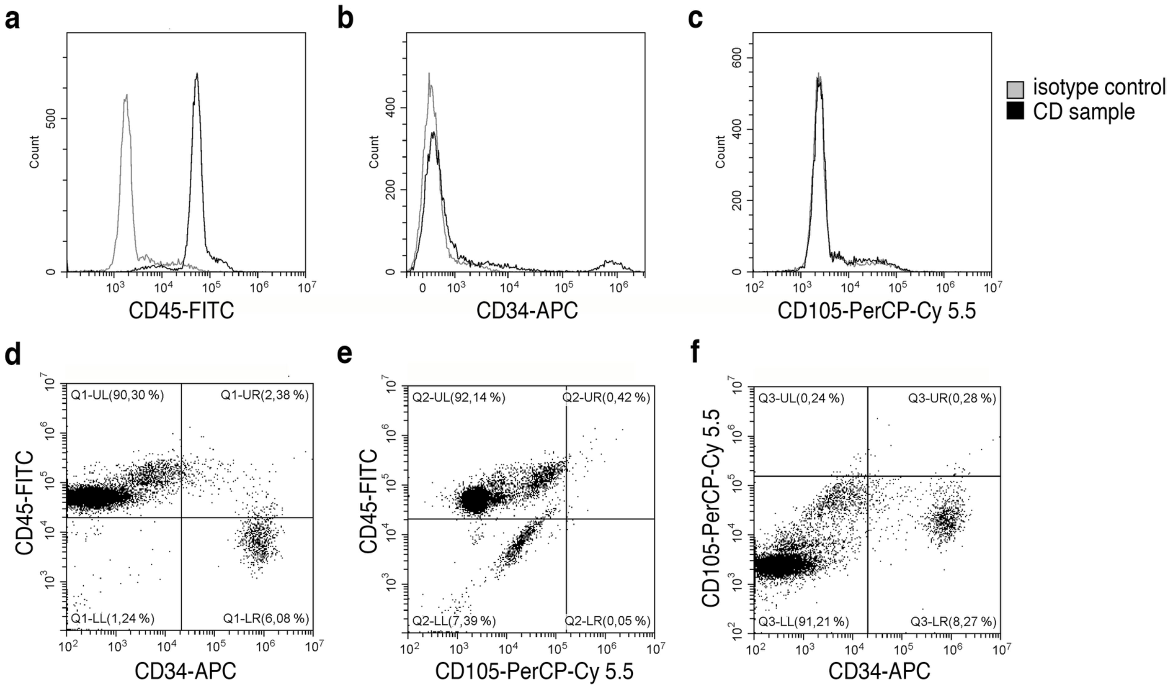

Supplementary Fig. 1. Immunophenotypic analysis of SVF cells isolated from fresh human subcutaneous adipose tissue biopsies. Flow cytometry analysis (FACS) of the stem cells markers CD34 (APC) and CD105 (PerCP-Cy 5.5). CD45 (FITC) was used for measurement of non-relevant hematopoietic cells due to blood contamination of the excised tissue biopsies. (**a-c**) Representative FACS histograms describing the expressions of CD45, CD34 and CD105. Isotype antibodies were used according to the manufacturer's recommendations for each antibody. (**d-f**) FACS analysis of cellular subsets in the SVF. All together 17 independent individuals were analysed.

Supplementary Fig. 2, Related to Fig. 3

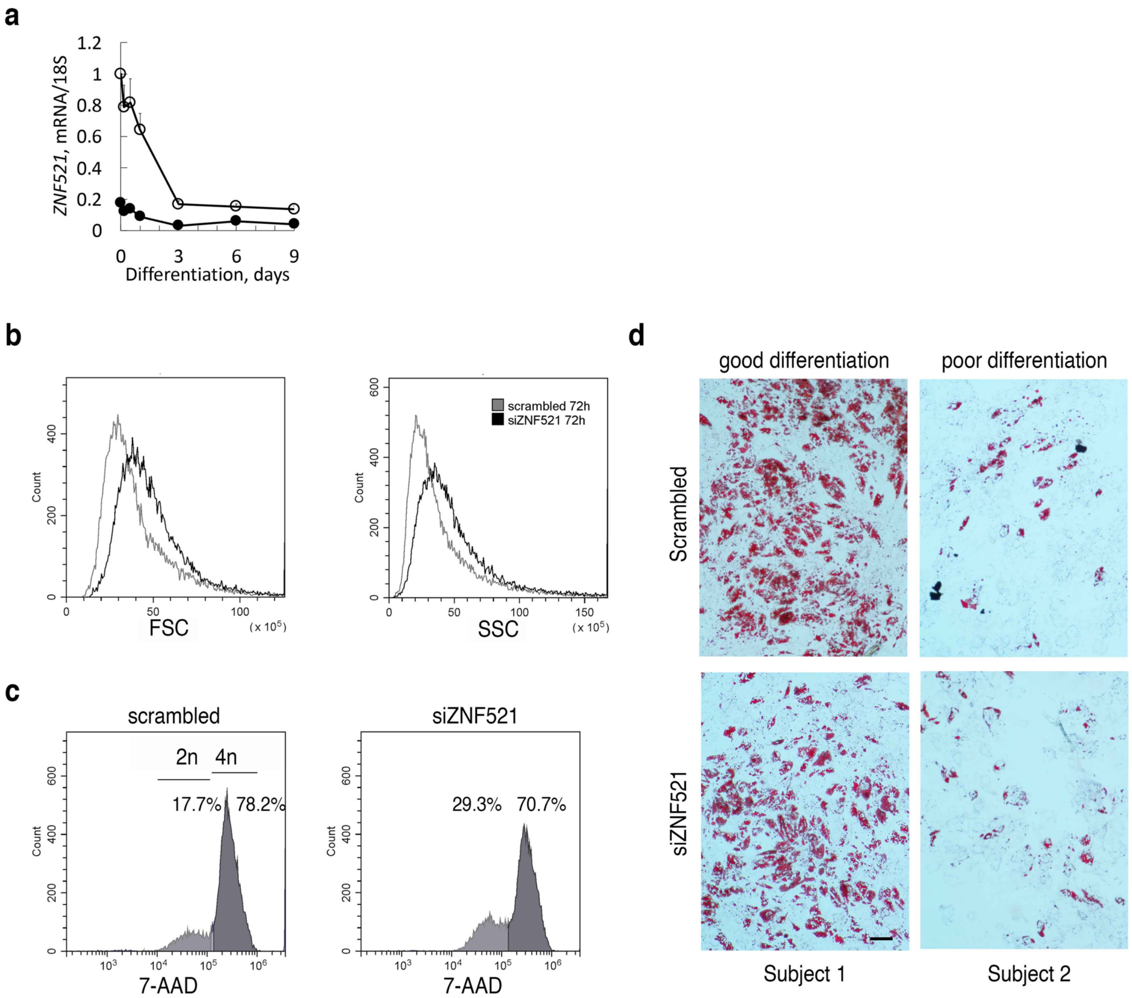

Supplementary Fig. 2. Immunofluorescence staining of incorporated 7-amino-actinomycin D (7-AAD) and FACS analysis of ZNF521 silenced cells. (**a**) Silencing ZNF521 reduced *ZNF521* expression with ≈88% at time 0h. Scrambled (control) samples open rings and siZNF521 closed rings. Control samples from Figure 1E. (**b**) Silencing ZNF521 reduced proliferation of the SVF cells with 30% (±4.5%, n=3 biological independent samples). Both forward scatter (FSC, cell size) and side scatter (SSC, granularity) increased in the ZNF521 silenced cells. SVF cells in passage 3 were used. Analysis was performed 72h after silencing of ZNF521. (**c**) Cells that actively synthesize DNA incorporate 7-AAD and progress through the S phase of the cell cycle. Silencing ZNF521 induced a decrease in DNA synthesis measured as a reduced 7-AAD content (4n). (**d**) Silencing ZNF521 did not markedly affect lipid accumulation during differentiation. Differentiation was started 72h after silencing of ZNF521 and ORO staining was performed at differentiation day 9. Results from one individual with good differentiation (Subject 1) and one with low differentiation capacity (Subject 2). Scale bar 100 μm.

Supplementary Fig. 3, Related to Fig. 4

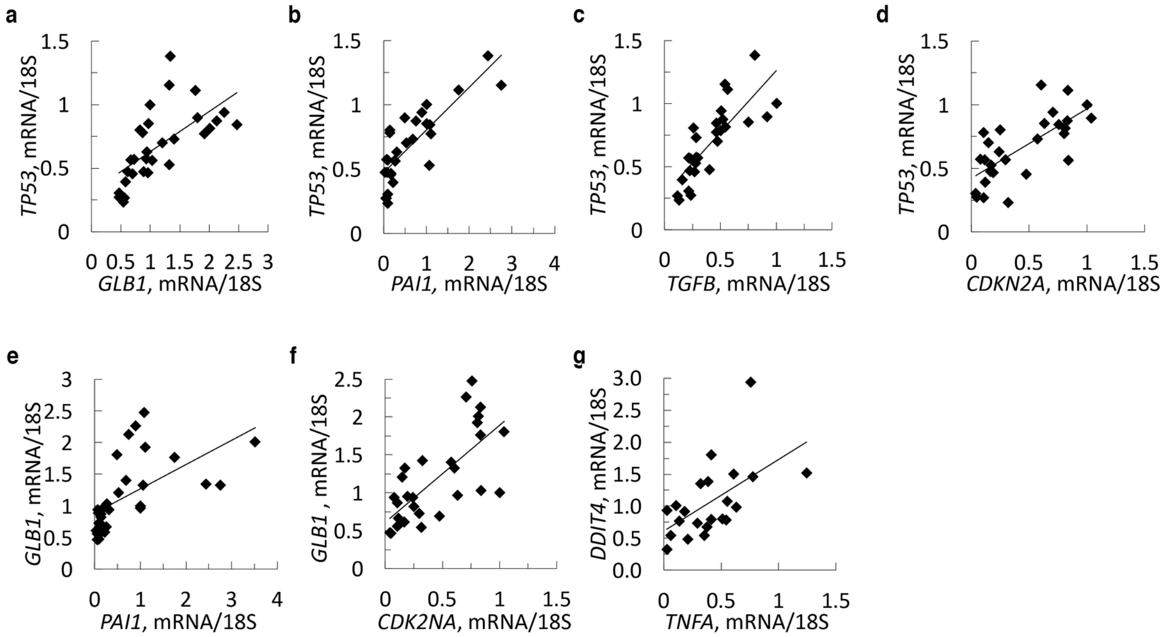

Supplementary Fig. 3. Correlation between selected senescence markers in intact SAT biopsies and differentiated SVF cells. (**a-d**) All senescence markers in intact biopsies correlated with *TP53*. *TP53* versus *GLB1, PAI1, CDKN2A/P16*^{INK4}*, TGFB1* and *GLB1.* Comparisons were made with Spearman's correlation coefficient. $P < 0.001$, n=28 biological independent samples. (e-**f**) *GLB1* versus *PAI1* and *GLB1* versus *CDKN2A/P16* ^{INK4} $P < 0.001$, n=28 biological independent samples. Comparisons were made with Spearman's correlation coefficient. mRNA expression was first normalized to 18S and then normalized to one individual (=1). (**g**) *DDIT4* is closely correlated with *TNFA* in differentiated cells, $P < 0.01$, n=22 biological independent samples. mRNA expression was first normalized to 18S and then normalized to expression in undifferentiated sample (=1).

Supplementary Fig. 4, Related to Fig. 4f

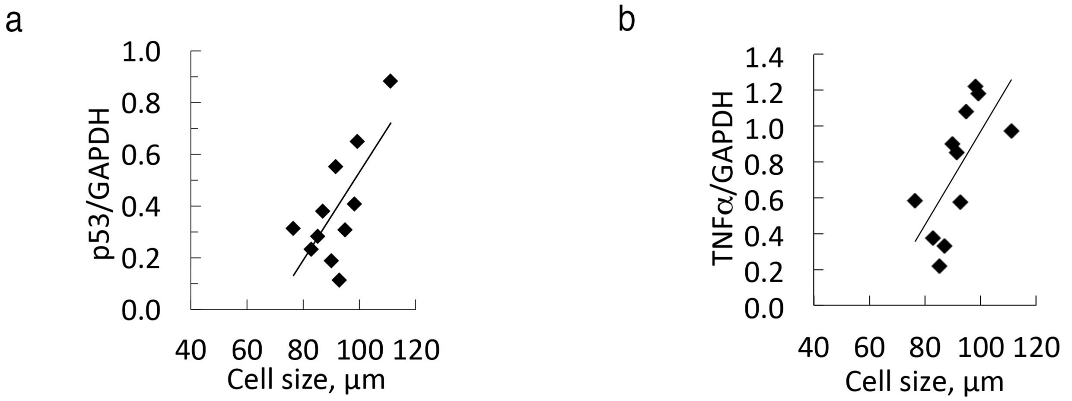

Supplementary Fig. 4. Correlation of p53 and TNFα with cell size in intact adipose tissue biopsies. Quantification of Western blots from Figure 4F. A total of 11 individuals were analysed. (**a**) Correlation between p53 and cell size, P 0.01, n=11 biological independent samples. (**b**) Correlation between TNFα and cell size, $P < 0.05$, n=11 biological independent samples. p53 and TNFα were first normalised to GAPDH before comparison with cell size. Comparisons were made with Spearman's correlation coefficient.

Supplementary Fig. 5

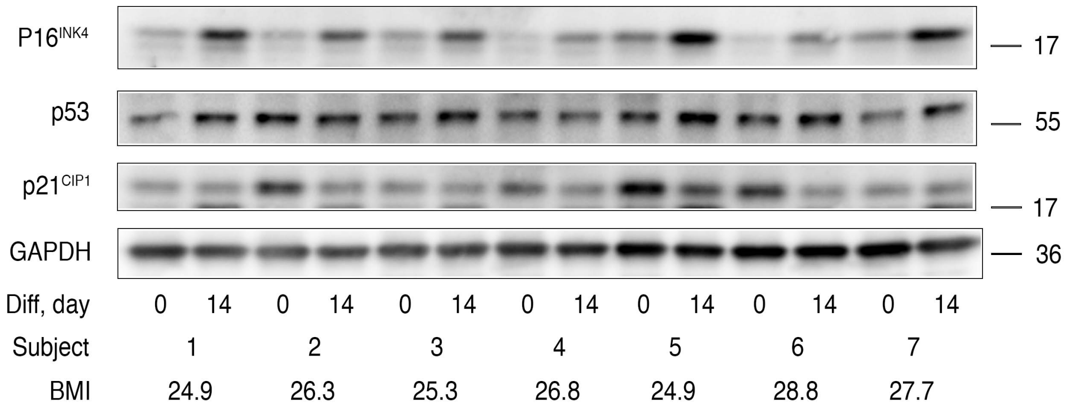

Supplementary Fig. 5. Increased susceptibility to develop cell senescence is linked to retained p53 and P16^{INK4} levels in differentiated adipocytes in FDR. Immunoblots showing P16^{INK4}, p53 and p21^{CIP1} from 7 FDR. Differentiation day 0 (SVF cells) and differentiation day 14 (mature adipocytes).

Supplementary Fig. 6.

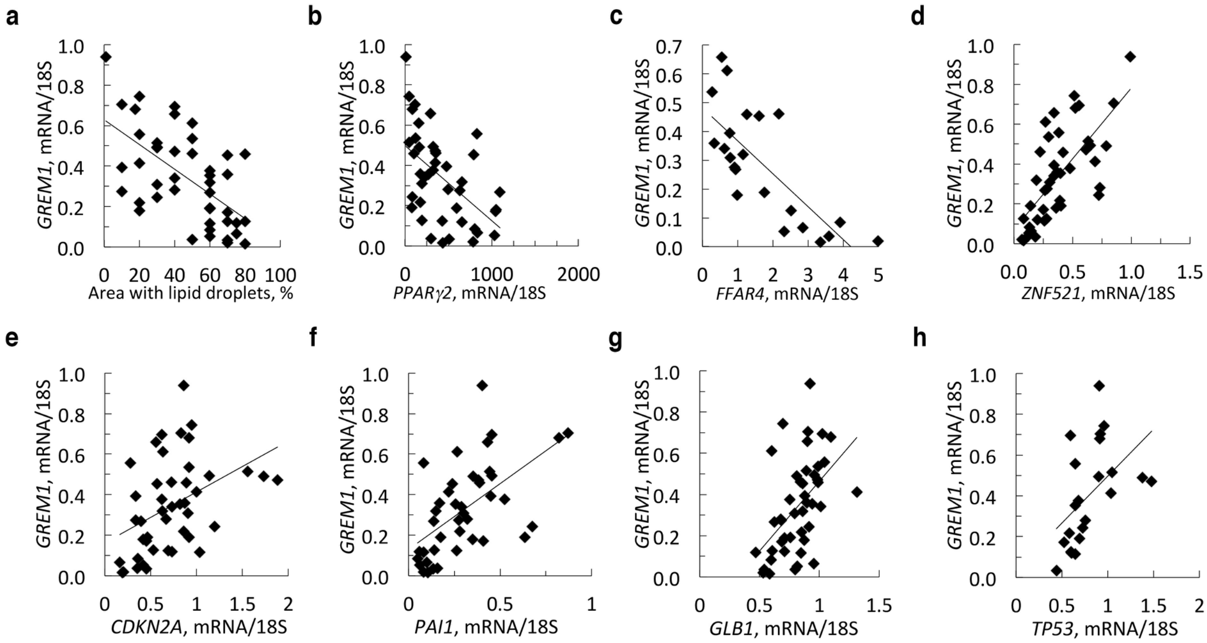

Supplementary Fig. 6. The BMP4 antagonist *GREM1*, highly expressed in cells with poor adipogenesis, correlated with markers of senescence. (**a-c**) *GREM1* is negatively correlated with lipid accumulation *P*<0.001, n=41 n=28 biological independent samples, induction of *PPARG2 P* < 0.00, n=41 n=28 biological independent samples, and *FFAR4 P* < 0.001, n=22 n=28 biological independent samples. (**d**) *GREM1* correlated closely with *ZNF521* as marker of undifferentiated cells *P* < 0.001, 41 n=28 biological independent samples. (**e-h**) *GREM1* correlated also with markers of senescence, *CDKN2A P* 0.002, n=41 biological independent samples, *PAI1 P* < 0.001, n=41 biological independent samples, *GLB1 P* < 0.001, n=22 biological independent samples and *TP53 P* = 0.003, n=22 biological independent samples. Spearman´s correlation coefficient was used since *ZNF521* was not normally distributed. Human SVF cells from subcutaneous adipose tissue were differentiated for 14 days. mRNA results were first normalized to 18S and then normalized to expression levels in the undifferentiated sample at 0h (=1).

Supplementary Fig. 7

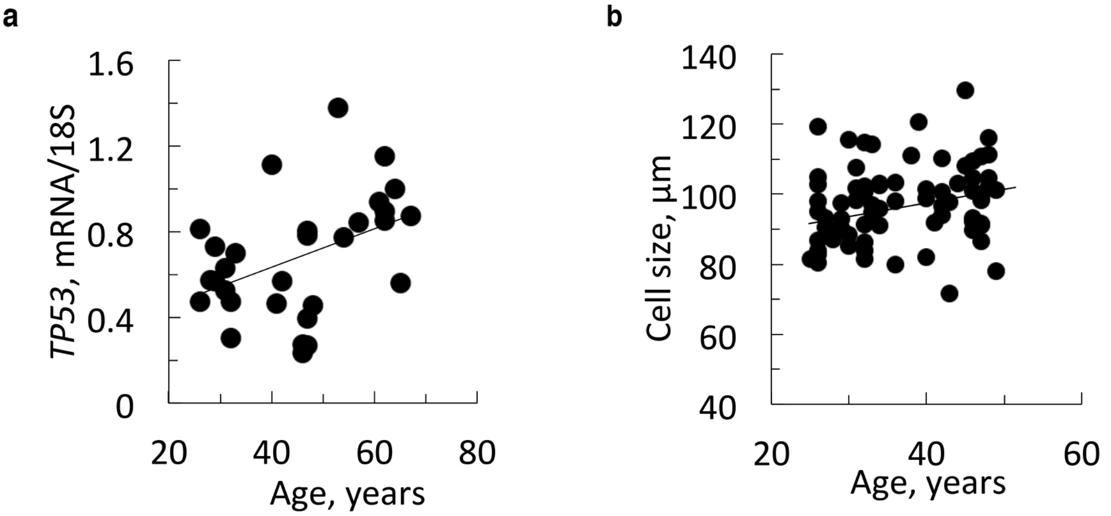

Supplementary Fig. 7. Donor age is correlated both with *TP53* and SAT cell size adjusted for BMI. (**a**) *TP53* is correlates with age in SAT tissue biopsies, $P < 0.05$, n = 31 biological independent samples. Range 26 – 67 years. (**b**) Cell size increases with age. R = 0.26, $P = 0.023$, n = 74 biological independent samples. Range (age) 25 – 49 years and cell size 71.5 – 129.6 µm. Data was also adjusted for BMI, R = 0.25, $P = 0.031$. (**a-b**) Spearman´s correlation coefficient was used since age was not normally distributed.

Supplementary Fig. 8

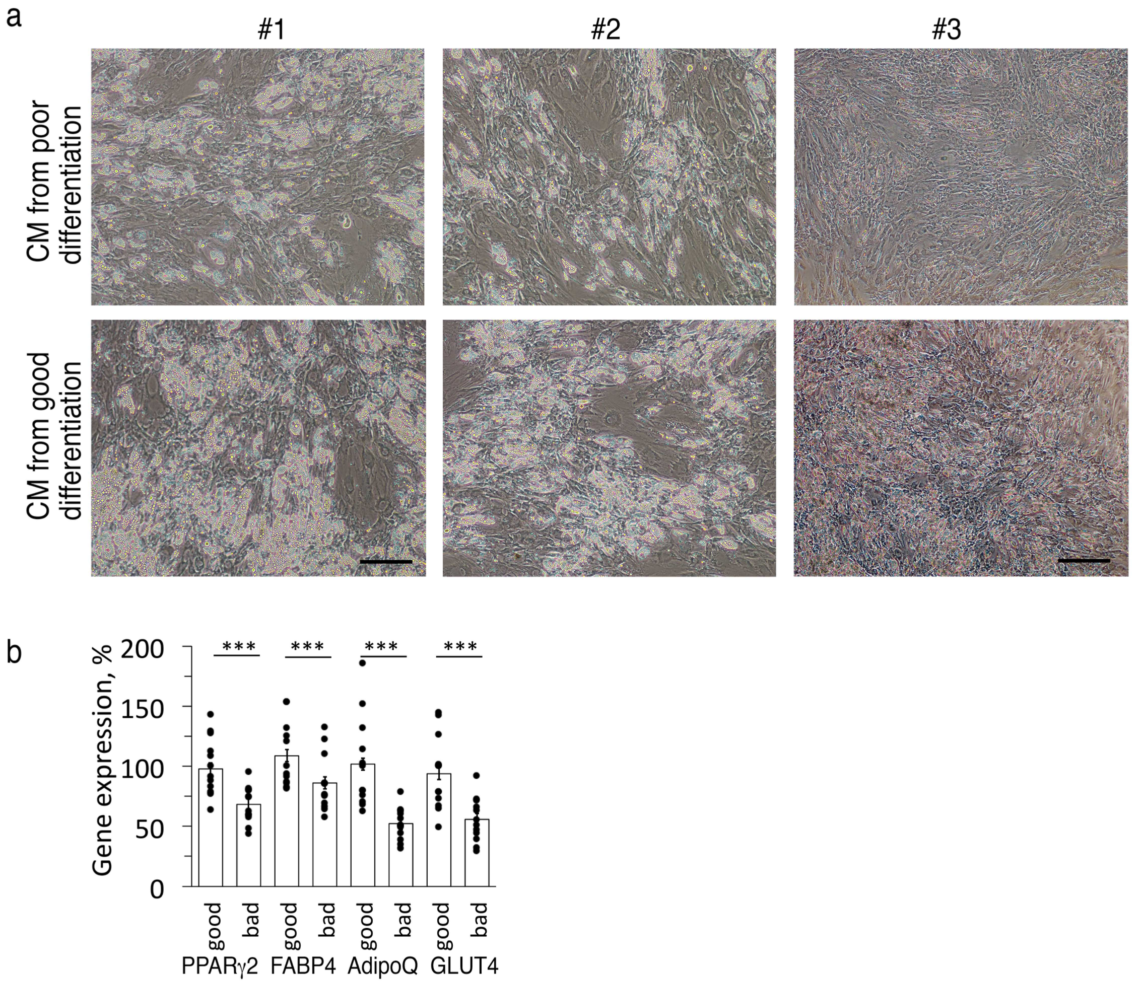

Supplementary Fig. 8. Conditioned medium from cells with poor differentiation (PD) lead to reduced adipogenesis compared to conditioned medium from cells with good differentiation (GD). Human adipogenic progenitor cells were differentiated with CM from 3 individuals with poor adipogenesis and 3 individuals with good adipogenesis. (a) Photographs show lipid accumulation of cells cultured with medium from PD (top) and medium from GD (below) at differentiation day 12. Magnification shown under the figures. Scale bars #1 and #2; 100 µm and #3; 200 µm. (b) Gene expression of *PPARγ2, FABP4*, *adiponectin* (*AdipoQ*) and *GLUT4*. Gene expression was first normalised to 18S, then to control cells of each individual cultured with regular medium at day 12. The individual with gene expression close to mean value of good differentiation was set to 100%. T-test was used for statistical correlation. ***$P < 0.001$; n = 12 biological independent samples.

Supplementary Table 1, related to Fig. 1 and Supplementary Fig. 1. Frequencies of progenitor/precursor cells subsets in subcutaneous adipose tissue biopsies. Results from FACS analysis. Results are a compilation of the results in Fig. 1.

	CD105	CD105/CD34	CD34	CD45	unstained
mean, %	0.02	0.96	21.66	73.05	4.31
sem	0.01	0.18	2.50	3.08	1.22
n	17	17	17	17	17

Supplementary Table 2, related to Fig. 4. Biometric data and expression of genes related to senescence of subjects in Fig. 4a-e.

	age		BMI		cell size			HOMA			
	mean	SD	mean	SD	mean	SD	n	index	SD	range	n
lean contr	38	±9.1	24.0	±3	90.2	±5.3	9	0.86	0.38	0.17-1.28	11
obese	37.3	±8.1	32.5	±4.2	105.7	±7.4	8	3.00	1.67	1.28-6.22	10
obese T2D	60.7	±4.6	33.1	±3.3	114.6	±8	10	-	-	-	-

	HOMA
	P
lean vs. obese	0.0012

	TP53		*PAI1*		*GLB1*		
	mean	SD	mean	SD	mean	SD	n
lean contr	0.47	±0.21	0.10	±0.04	0.73	±0.20	9
obese	0.62	±0.24	0.88	±1.12	1.18	±0.50	8
obese T2D	0.92	±0.23	1.18	±0.79	1.63	±0.56	10

	TP53	*PAI1*	*GLB1*
	P	*P*	*P*
obT2D vs. lean	0.0002	0.0004	0.0001
obese vs. lean	0.1876	0.0416	0.0171

Gene expression was analysed in intact human SAT biopsies i.e. a mixture of adipocytes, progenitor, precursor and haematological cells. mRNA results were first normalized to 18S and then normalized to expression levels in one individual (=1). Comparisons were made with Spearman's correlation coefficient.

Supplementary Table 3, related to Fig. 4. Expression of senescence markers in differentiated adipocytes and their correlations with BMI and markers of adipogenesis.

	**versus**	**n**	**R**	***P***	**corr.**
GLB1	*ZNF521*	43	0.5134	<0.001	pos.
	Oil Red 0	44	0.4333	<0.01	neg.
	FFAR4	22	0.6178	<0.01	neg.
	PPARG2	42	0.2703	ns	-
	BMI	44	0.2149	ns	-
TP53	*ZNF521*	22	0.6415	<0.01	pos.
	Oil Red 0	22	0.4996	<0.05	neg.
	FFAR4	22	0.5660	<0.01	neg.
	PPARG2	20	0.4299	<0.05	neg.
	BMI	22	0.2743	ns	-
CDKN2A	*ZNF521*	42	0.6584	<0.001	pos.
	Oil Red 0	42	0.3497	<0.001	neg.
	FFAR4	22	0.4380	<0.05	neg.
	PPARG2	42	0.6106	<0.001	neg.
	BMI	44	0.2442	ns	-
PAI1	*ZNF521*	39	0.6677	<0.001	pos.
	Oil Red 0	39	0.5109	<0.001	neg.
	FFAR4	20	0.6884	<0.001	neg.
	PPARG2	37	0.5432	<0.001	neg.
	BMI	39	0.4053	<0.01	pos.
TGFB1	*ZNF521*	22	0.5108	<0.05	pos.
	Oil Red 0	22	0.2854	ns	-
	FFAR4	22	0.6599	<0.001	neg.
	PPARG2	20	0.6685	<0.001	neg.
	BMI	22	0.2544	ns	-
GLB1	*PAI1*	41	0.4480	<0.001	pos.
	CDKN2A	44	0.4274	<0.01	pos.

Isolated SVF cells from SAT biopsies were differentiated for 14 days. mRNA levels were first normalized to 18S and then normalized to expression in undifferentiated cells at 0h, before initiation of differentiation. Ns = no significance.

Supplementary Table 4. Antibodies used for Western blot, immunofluorescence and FACS analysis.

Primary antibodies, Western blot

- ZNF521, (1:750) Sigma-Aldrich, SAB3500840, lot number 465108031
- GAPDH, (1:3000) Santa Cruz (0411): sc-47724
- P16^{INK4}, (1:500) Abcam (1D7D2A1): ab201980, lot number GR286368-7
- mTOR, (1:500) Cell Signaling (L27D4): #4517
- Rb, (1:500) Santa Cruz (C-15): sc-50
- p53, (1:500) Cell Signaling (1C12): #2524
- p21^{CIP1}, (1:500) Santa Cruz (F-5): sc-6246
- BMP4, Abcam: (1:1000) 39973, lot number GR194662-1
- β-tubulin, (1:2500) Cell Signaling (9F3): #2128
- ZNF423, (1:500) Santa Cruz, OAZ (H-105): sc-48785
- Lamin A/C, (1:500) Santa Cruz (N-18): sc-6215
- TNFα, (1:500) Cell Signaling: #6945 (D5G9), lot 4

Secondary antibodies, Western blot

- Horse anti-mouse IgG, (1:2000) HRP-linked, Cell signalling #7076
- Goat anti-rabbit IgG, (1:2000) HRP-linked, Cell signalling #7074
- Donkey anti-goat IgG, (1:1000) HRP-linked, Santa Cruz sc-2020

FACS analysis

- CD105, (1:40) BD 560819, PerCP-Cy 5.5, Clone 266
- CD34, (1:20) BD 560940, APC, Clone 581
- CD45, (1:20) BD 560976, FITC, Clone H130
- Isotype control, (1:40) PerCP-Cy 5.5, BD 550795, Clone MOPC-21
- Isotype control, (1:20) APC, BD 555751, Clone MOPC-21
- Isotype control, (1:20) FITC, BD 555748, Clone MOPC-21

Supplementary Table 5. Probes and primers used for quantitative real-time PCR.

Assay on Demands, ThermoFisher Scientific

Gene	assay number	Gene	assay number
ZNF521	Hs00296682_m1	*FFAR4*	Hs00699184_m1
ATGL	Hs00386101_m1	*ChREBP*	Hs00263027_m1
FASN	Hs00188012_m1	*GLB1*	Hs01035168_m1
TP53	Hs01034249_m1	*CDKN2A*	Hs00923894_m1
TGFB1	Hs00171257_m1	*GREM1*	Hs01879841_s1
DDIT4	Hs0111686_g1		

In house design

Gene		Sequence (5'-3')
C/EBPB	Forward	AGCACAGCGACGAGTACAAGATC
	Reverse	TGTCGCGGCTCTTGCG
	Probe	CGCGAGCGCAACAACATCGC
C/EBPD	Forward	GCCCCGAGTACCGGCA
	Reverse	TGTCGCGGCTCTTGCG
	Probe	CGCGAGCGCAACAACATCGC
C/EBPA	Forward	CCAAGAAGTCGGTGGACAAGA
	Reverse	CGCACCGCGATGTTGTT
	Probe	CGCCGCACCCGGTACTCGTT
PPARG2	Forward	TCTGCAAACATATCACAAGAAATGA
	Reverse	GTCTTCCATTACGGAGAGATCCA
	Probe	CCACCAACTTTGGGATCAGCTCCG
ADIPOQ	Forward	CCGGGCCATAATGGGG
	Reverse	CCCTTAGGACCAATAAGACCTGG
	Probe	TGCCATCTCTGCCATCACGGCC
BMP4	Forward	GAGCTTCCACCACGAAGAACA
	Reverse	GGGATGCTGCTGAGGTTAAAGA
	Probe	TGGAGAACATCCCAGGGACCAGTGA
FABP4	Forward	GACAGGAAAGTCAAGAGCACCATA
	Reverse	GACGCATTCCACCACCAGTT
	Probe	CTGCACATGTACCAGGACACCCCCA
GLUT4	Forward	TCTGGCATCAATGCTGTTTTCTAT
	Reverse	ACCAACAACACCGAGACCAAG
	Probe	TGACCACACCAGCTCCTATGGTGGC
PAI1	Forward	TCCTCATCCACAGCTGTCATAGT
	Reverse	GGGTCTGTCCATGATGATCTCC
	Probe	TCAGCCCGCATGGCCCC
