# Supplementary material for: Reduced subcutaneous adipogenesis in human hypertrophic obesity is linked to senescent precursor cells
Source: Nat Commun. 2019 Jun 21;10:2757. doi: 10.1038/s41467-019-10688-x (PMC6588633; doi:10.1038/s41467-019-10688-x)
Supplement: Supplementary file 2 — Reporting Summary [file 41467_2019_10688_MOESM2_ESM.pdf]

nature research

Corresponding author(s): Birgit Gustafson, Annika Nerstedt and Ulf Smith

Reporting Summary

Nature Research wishes to improve the reproducibility of the work that we publish. This form provides structure for consistency and transparency in reporting. For further information on Nature Research policies, see Authors & Referees and the Editorial Policy Checklist.

Statistical parameters

When statistical analyses are reported, confirm that the following items are present in the relevant location (e.g. figure legend, table legend, main text, or Methods section).

n/a	Confirmed	
☐	☒	The exact sample size (*n*) for each experimental group/condition, given as a discrete number and unit of measurement
☐	☒	An indication of whether measurements were taken from distinct samples or whether the same sample was measured repeatedly
☐	☒	The statistical test(s) used AND whether they are one- or two-sided<br>*Only common tests should be described solely by name; describe more complex techniques in the Methods section.*
☐	☒	A description of all covariates tested
☐	☒	A description of any assumptions or corrections, such as tests of normality and adjustment for multiple comparisons
☐	☒	A full description of the statistics including central tendency (e.g. means) or other basic estimates (e.g. regression coefficient) AND variation (e.g. standard deviation) or associated estimates of uncertainty (e.g. confidence intervals)
☐	☒	For null hypothesis testing, the test statistic (e.g. *F*, *t*, *r*) with confidence intervals, effect sizes, degrees of freedom and *P* value noted<br>*Give P values as exact values whenever suitable.*
☒	☐	For Bayesian analysis, information on the choice of priors and Markov chain Monte Carlo settings
☒	☐	For hierarchical and complex designs, identification of the appropriate level for tests and full reporting of outcomes
☒	☐	Estimates of effect sizes (e.g. Cohen's *d*, Pearson's *r*), indicating how they were calculated
☐	☒	Clearly defined error bars<br>*State explicitly what error bars represent (e.g. SD, SE, CI)*

Our web collection on statistics for biologists may be useful.

Software and code

Policy information about availability of computer code

Data collection	Statistical analysis were performed with IBM SPSS Statistics version 23 except for HOMA-IR were the Homa2 calculator was used. Radcliff, Dep. of Medicine, University of Oxford, UK, www.dtu.ox.ac.uk/homacalculator/. For FACS analysis was CytExpert 1.2 (Beckman Coulter) used. Histology images were photographed by Zeiss AxioCam MRM or NIKON Eclipse 100 microscope cameras.
Data analysis	Western blotting Image quantification was performed with Image Lab v5 and v6 (BioRad). Data analysis was performed with PASWstatistics (SPSS Inc.) for Macintosh.

For manuscripts utilizing custom algorithms or software that are central to the research but not yet described in published literature, software must be made available to editors/reviewers upon request. We strongly encourage code deposition in a community repository (e.g. GitHub). See the Nature Research guidelines for submitting code & software for further information.

Data

Policy information about availability of data

All manuscripts must include a data availability statement. This statement should provide the following information, where applicable:

- Accession codes, unique identifiers, or web links for publicly available datasets
- A list of figures that have associated raw data
- A description of any restrictions on data availability

All data presented in this manuscript are included in the main and supplementary figures. Uncropped and unprocessed Western blots are found int the Source Data file.

Field-specific reporting

Please select the best fit for your research. If you are not sure, read the appropriate sections before making your selection.

☒ Life sciences ☐ Behavioural & social sciences ☐ Ecological, evolutionary & environmental sciences

For a reference copy of the document with all sections, see nature.com/authors/policies/ReportingSummary-flat.pdf

Life sciences study design

All studies must disclose on these points even when the disclosure is negative.

Sample size	Samples sizes were chosen based on previous experience in our laboratory.
Data exclusions	No data was excluded.
Replication	If similar results were obtained from at least 3 independent experiments, replication was considered as successful.
Randomization	The experiments were performed and analyzed in non-randomized fashions.
Blinding	The experiments were performed and analyzed in non-blinded fashions.

Reporting for specific materials, systems and methods

Materials & experimental systems

n/a	Involved in the study	
☐	☒	Unique biological materials
☐	☒	Antibodies
☒	☐	Eukaryotic cell lines
☒	☐	Palaeontology
☒	☐	Animals and other organisms
☐	☒	Human research participants

Methods

n/a	Involved in the study	
☒	☐	ChIP-seq
☐	☒	Flow cytometry
☒	☐	MRI-based neuroimaging

Unique biological materials

Policy information about availability of materials

Obtaining unique materials	Human adipose tissues and stromal cell fractions are highly limited. Materials might be available from the authors upon request.

Antibodies

Antibodies used	All antibodies are listed in Supplementary Table 5. IF: ZNF521, Sigma-Aldrich, HPA023056, lot number A104071, Western blot: ZNF521 Sigma-Aldrich, HPA023056, lot number A104071; GAPDH Santa Cruz (0411): sc-47724; P16INK4 Abcam (1D7D2A1): ab201980, lot number GR286368-7; mTOR Cell Signaling (L27D4): #4517; Rb Santa Cruz (C-15): sc-50; p53 Cell Signaling (1C12): #2524; p21CIP1 Santa Cruz (F-5): sc-6246; BMP4 Abcam: 39973, lot number GR194662-1; β-tubulin Cell Signaling (9F3): #2128; ZNF423 Santa Cruz OAZ (H-105): sc-48785; Lamin A/C Santa Cruz (N-18): sc-6215; TNFa Cell Signaling #6945. FACS: CD105 BD 560819; PerCP-Cy 5.5 Clone 266; CD34 BD 560940 APC Clone 581; CD45 BD 560976 FITC Clone H130. Isotype control PerCP-Cy

5.5, BD 550795, Clone MOPC-21; Isotype control APC, BD 555751, Clone MOPC-21; Isotype control FITC, BD 555748, Clone MOPC-21.

Validation

All antibodies were validated for use on the manufacturer's website. Antibodies for FACS anlysis were analyzed with a flow cytometer from Beckman Coulter (Cytoflex) and data were analyzed with CytExpert software (Beckman Coulter). Prior to use the antibodies and isotype controls were validated and titrated using cultured human stromal cells and fresh stromal cells isolated from human subcutaneous adipose tissue.

Human research participants

Policy information about studies involving human research participants

Population characteristics

Material used for RNA and Western blots consists of 44 individuals F/M 10/31 and 3 unknown gender and used for RNA and Western blots: Mean body mass index (BMI) was 27.7+/-9.1 kg/m2 (means+/-SD; range 20.6-36.1). Cell size 98.3+/-11.7 µm (mean +/-SD; range 73.7-129.6). Age 42+/-10.7 years (mean+/-SD; range 22-69). Material 2 (RNA) : Mean body mass index (BMI) was 29.9+/-5.5 kg/m2 (means+/-SD; range 18.1-39.5). Cell size 103.1+/-13.9 µm (mean +/-SD; range 78-129.6). Age 45+/-13 years (mean+/-SD; range 26-67).

Recruitment

The individuals were recruited to laboratory through newspaper advertisement and were a part of other on-going studies at laboratory. Human subcutaneous adipose tissue was obtained by needle biopsy.

Flow Cytometry

Plots

Confirm that:

☒ The axis labels state the marker and fluorochrome used (e.g. CD4-FITC).

☒ The axis scales are clearly visible. Include numbers along axes only for bottom left plot of group (a 'group' is an analysis of identical markers).

☒ All plots are contour plots with outliers or pseudocolor plots.

☒ A numerical value for number of cells or percentage (with statistics) is provided.

Methodology

Sample preparation

Stromal cell fraction from human subcutaneous adipose tissue was obtained by needle biopsy and cells were isolated with collagenase digestion. Erythrocytes were removed with BD Lysing solution (BD555899). The stromal fraction was washed extensively with PBS 2% BSA and stained for 30 min at 4°C. All antibodies were directly conjugated and no fixation or permeabilization was performed.

Instrument

Beckman Coulter CytoFlex pn:B50952 B3-R3-V0

Software

CytExpert 1.2

Cell population abundance

In each experiment at least 10,000 events were acquired.

Gating strategy

The cells were serially gated as follows: FSC-A/SSC-A were used as an initial gate for cell debris and aggregates with unstained cells, followed by isotype controls and stained cells, CD45/FITC against CD34/APC, and CD34/APC against CD105/PerCP 5.5. Representative flow cytometry plots are provided to show all subsequent gating steps.

☒ Tick this box to confirm that a figure exemplifying the gating strategy is provided in the Supplementary Information.
